# Impact of single-use technology on continuous bioprocessing

**DOI:** 10.1186/1753-6561-7-S6-P39

**Published:** 2013-12-04

**Authors:** William G Whitford, Brandon L Pence

**Affiliations:** 1 Thermo Fisher Scientific, 925 West 1800 South, Logan, Utah 84321, USA

## Background

**Figure 1:**
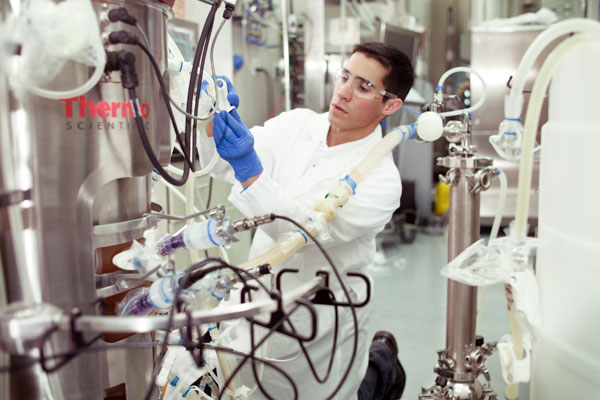
**Hybrid intensified perfusion-based continuous bioproduction in a Thermo Scientific HyPerforma S.U.B**. TK 250L supported by the Refine Technology ATF System.

Single-use (SU) technologies supply a number of values to any mode of bioprocessing, but can provide some specific and enabling features in continuous bioprocessing (CB) implementations [1-3]. Most every operation in a CB process train is now supported by a commercially available single-use, or at least hybrid, solution (Figure 1). First of all, many of the SU equipment and solutions being developed for batch bioproduction have the same or related application in CB systems. Examples here include simple equipment such as tubings and connectors, to more complex applications such as the cryopreservation of large working stock aliquots in flexible bioprocess containers (BPCs). The list of CB-supporting SU technologies being developed is large and growing.

## Results

A SU advantage in process development is its supports of an open architecture approach and a number of hybrid designs. Such designs include combining reusable and single-use systems, or between divergent suppliers of particular equipment. Especially in bioproduction, the many flexibilities of SU support a manufacturing platform of exceptional efficiency, adaptability, and operational ease. Advances designs in SU transfer tubing, manifold design and container porting also supports creativity in process design. This is of particular value in designing a process with such demands as entirely new flow paths or lot designations, such for CB.

SU systems upstream provide a reduced footprint and eliminate of the need for cleaning and sterilization service. This complements perfusion culture's inherently smaller size and independence from cleaning for extended periods of time.

Several newer approaches to formulating process fluids support the concept of CB. Single-use mixing systems are typically constructed of a rigid containment system with a motor and controls driving radiation-sterilized single-use bags equipped with disposable impeller assemblies. From a variety of manufacturers there are a number of distinct approaches to motor/disposable impeller assembly linkages, tubing lines and connections. Also appearing are a number of exciting SU sampling, sensing, and monitoring solutions. Single-use powder containers permit seamless transfer between powder and liquid formulation steps, and the ridged mixing containers are available in jacketed stainless steel for heating and cooling requirements. Surprisingly, the "topping-up" of large-scale single-use fluid containers with newly prepared buffer to provide a virtually unlimited and constant supply of each buffer/media type can be validated for GMP manufacturing procedures.

Process flexibility is a key feature in both SU and CB. CB contributes to overall process flexibility in that equipment tends to be easy to clean, inspect and maintain − and generally promotes simple and rapid product changeover. SU systems can provide similar flexibility and ease product changeover because they tend to be more modular and transportable than much of the older batch equipment. In fact the size, configuration and reduced service requirements of SU systems actually encourage diversity of physical location within a suite or plant, as well as re-location to other manufacturing sites.

Due to its inherent demand for immediate process data and control capabilities, CB supports initiatives in continuous quality verification (CQV), continuous process verification (CPV), and real-time release (RTR). Although CB will not be feasible for all products and processes, many implementations well-support a "platform" approach, in which a single process supports more than one product. CB most always shortens the process stream, reduces downtime, and greatly reduces handling of intermediates. These features complement the operational efficiencies of SU systems, contributing to a greatly reduced cumulative processing time for the API. Furthermore, they greatly simplify production trains and inherently facilitate application of closed processing approaches to individual operations and even processes. Especially in bioproduction, the modularity and integral gamma irradiation sterility of SU combined with the sustained operation of CB promise the appearance of platforms of unparalleled operational simplicity and convenience.

The heart of a CB approach is the bioreactor. Perfusion bioreactors have been successfully employed in bioproduction, even biopharmaceutical production, for decades. And, rather remarkably, disposable bioreactors have been available for nearly 20 years. At the research scale there have even been single-use hollow fiber perfusion bioreactors available from a variety of vendors for over 40 years. However, only recently have commercially available SU and hybrid production-scale perfusion-capable equipment become available.

The production-scale CB enabling SU bioreactor technologies now becoming commercial available include single-use and hybrid perfusion-capable reactors (Figure 1); a growing variety of SU and hybrid monitoring probes and sensors; SU pumps and fluid delivery automation of various design; and automated SU online sampling, interface, valving and feeding technologies. Their coordinated implementation in actual production settings with appropriate control is now beginning.

Justified or not, concerns in the implementation of CB include performance reliability (incidence of failure), validation complexity, process control and economic justification. But for many processes, such previous limitations -- or their perception -- are being alleviated by advances in CB processing technology and OpEx driven advances bioprocess understanding, reactor monitoring and feedback control. However, while some CB attributes inherently provide immediate advantages (such as reduces reactor residency time) others do present challenges (such as cell-line stability concerns).

Due to the limited contribution of API manufacturing to small-molecule pharmaceutical cost, the limited bottom-line financial savings of CB has been a concern. However, biopharma is a different animal in general, and as such trends as globalization and biosimilars alter the picture even further, the financial benefits of CB are becoming even stronger.

The fact that many SU systems are constructed of standards compliant and animal product-free materials supports CB applications in a wide variety of product types and classification. In fact, SU systems are available to most any process format (eg, microcarriers and suspension), platform (eg, cell line, vectors, culture media), mode (eg, dialysis or enhanced perfusion) or scale (eg, through rapid, inexpensive scale-out). "Futureproofing", or supporting the sustainability of a new CB process in the face of product lifecycle or emerging technology imperative, is supported by many SU features. Examples here include SUs low initial facility, service and equipment cost and especially SU's undedicated manufacturing suits and ease of process train reconfiguration.

## Conclusion

As advanced single-use solutions are applied to single-use perfusion mode-capable reactors, the design of integrated closed, disposable and continuous upstream bioproduction systems are finally being realized.
